# Ecological Basis for Rational Phage Therapy

**Published:** 2010-04

**Authors:** A.V. Letarov, A.K. Golomidova, K.K. Tarasyan

**Affiliations:** Winogradsky Institute of Microbiology, Russian Academy of Sciences

**Keywords:** bacteriophages, phage therapy, human body microbiota, animal body microbiota, bacteriophage ecology

## Abstract

Understanding the mutual interactions of bacterial and phage populations in the environment of a human or animal body is essential in any attempt to influence these complex processes, particularly for rational phage therapy. Current knowledge on the impact of naturally occurring bacteriophages on the populations of their host bacteria, and their role in the homeostasis maintenance of a macro host, is still sketchy. The existing data suggest that different mechanisms stabilize phage–bacteria coexistence in different animal species or different body sites. The defining set of parameters governing phage infection includes specific physical, chemical, and biological conditions, such as pH, nutrient densities, host prevalence, relation to mucosa and other surfaces, the presence of phage inhibiting substances, etc. Phage therapy is also an ecological process that always implies three components that form a complex pattern of interactions: populations of the pathogen, the bacteriophages used as antibacterial agents, and the macroorganism. We present a review of contemporary data on natural bacteriophages occuring in human– and animal–body associated microbial communities, and analyze ecological and physiological considerations that determine the success of phage therapy in mammals.

## INTRODUCTION

The use of bacteriophages as antibacterial agents was first suggested by one of the scientists who discovered bacteriophages. F. D’Errele was the first to use a phage preparation to treat a severe case of child dysentery in 1919, only two years after the publication of his first article, which reported the discovery of bacterial viruses [1, 2]. The therapeutic use of phages in Western medicine spread widely in the late 30s–early 40s, but it was subsequently almost completely forgotten because of highly unstable results, which was due to insufficient development of phage biology, phage production methods, the storage and use of drugs, and on the other hand, to the triumph of antibiotic treatment of bacterial infections, which seemed a simpler and better solution to the clinical problems in that field [2]. Nevertheless, the development and use of therapeutic phage preparations never stopped in the Soviet Union, as well as in Poland and the Czech Republic. The vast experience accumulated in those countries indicates that phage–based drugs are effective and safe. Today, mass–production of such antibacterial drugs continues in Russia and Georgia (where F. D’Errele personally took part in the establishment of the Bacteriophage, Microbiology and Virology Institute (now named after G. Eliava) in the 1930s). In Poland therapeutic phage preparations are produced and used in a specialized center at the Institute of Immunology and Experimental Therapy in Wroclaw.

The continuing increase in occurence of drug–resistant strains of pathogenic microorganisms and the dramatic slump in the development and marketing of novel antibacterial agents have spurred renewed interest towards phage therapy in the last 20 years in both Russian and Western medicine [3, 4]. Several reviews on issues related to phage therapy have recently been published [2, 5, 6, 7].

Modern phage therapy mostly uses bacteriophages, which belong to the Caudovirales order (“tailed phages”). About 96% of all known phages belong to this group [8]. Tailed phages are divided into 3 families:

- Podoviridae, which have a short noncontractile tail;
- Siphoviridae, which have a long noncontractile tail; and
- Myoviridae, which have a contractile tail.

RNA–bearing phages from the Leviviridae family, as well as filamentous phages bearing a single–strand DNA belonging to Inoviridae family, are also of ecological importance. Leviviral virions have small (~26 nm) icosahedral particles, which encapsulate a single–strand RNA genome. The filamentous particles of inoviruses are molecules of circular single–strand DNA, which is covered by low–molecular–weight envelope proteins.

Members of the other prokaryote virus families (currently there are 14 families defined by ICTV) are rarely encountered and probably do not play a major role in the microecology of symbiotic populations in mammals. More details on the classification of bacteriophages and the biology of particular groups of these viruses can be found in [8] and [9].

Despite the fact that virulent phages (those which cannot integrate their genome into the bacterial genome and cannot form lysogenic strains) cause the death of all infected cells and, reproducing exponentially under certain conditions and killing a large number of sensitive bacteria, these viruses stably coexist with their hosts in practically all known natural ecosystems [10]. This coexistence has continued throughout the whole history of the life on Earth [11], and the interactions between bacteria and phages at the level of the population are fairly complex and multifaceted; thus, the simplistic interpretation of phages as the “natural enemies” of bacteria is inaccurate.

From the microbiologist’s point of view, an animal or human organism is a complex microcosm that includes several interconnected ecosystems located in various organs and regions of the body [12] and is inhabited by more or less dense populations of microorganisms, including bacteriophages [13].

The role of bacteriophages in the microflora of the body is considered to be “significant,” but in reality, knowledge on the subject is scarce. The presence of phages in normal body microflora was demonstrated by the discoverer of phages, Felix D’Errele (see above). He discovered enterobacterial phages in human and animal faeces [1]. Nevertheless, neither qualitative nor quantitative methods for measuring the interactions of phage and bacterial populations in symbiotic microbial systems have been developed for even a single animal species. The understanding of the peculiarities of ecologico–physiological interactions in the tri–partite system of “bacteria–phages–macroorganism” must form the theoretical groundwork for controlling this system, including the application of exogenous phage preparations which will eliminate unwanted bacterial populations.

This review analyzes the current ideas on the ecology of bacterial viruses in human and animal microbial systems, the mechanisms of direct interaction between endogenous and exogenously applied phage particles and between these phages and the cells and organs of a macroorganism. Also we briefly discuss the key theoretical principles underlying the therapeutic use of phage preparations.

## Bacteriophages in the normal microflora of a body

**The quantity and variety of phages in animal microflora**. The bulk of articles on this subject study bacteriophages inhabiting the digestive tract of mammals (see further), including the colon (and feces), the rumen (for ruminants) and forestomac (for marsupials) microflora [14], where phages are the most abundant free viral particles [13– 26]. Interestingly, most of the RNA–bearing viruses in human feces are plant viruses particles, which are ingested with food [27]. The presence of free viral particles in the lungs or on the skin of animals has yet to be established. Yet several pathological conditions, such as mucoviscidosis, are characterized by the simultaneous presence of phages and bacteria, including * Pseudomonas aeruginosa * [28]. Surprisingly, free virus particles have not been detected in the mouth [29].

Since the symbiotic populations of the human and animal gastro –intestinal tracts (GIT) include several hundred species (and thus up to a 1,000 strains) of bacteria and archaea, each of which is a potential target for phage infection, the majority of phages that inhabit the organism cannot be studied using culturing methods. However, the use of direct methods of analysis, such as extraction and purification of noncultutable viral populations from different regions of the GIT and their analysis by light and electron microscopy, electrophoretic separation and metagenomics, has allowed researchers to assess the representation and variety of phages in microbial populations. A detailed analysis of these studies is beyond the scope of this article; they are comprehensively reviewed in [13]. Notably, the concentration of free virus particles in the colon region and the rumen of ruminant animals is estimated at about 10^8^–10^11^ particles per milliliter. The number of distinct morphological types is estimated to range from tens to hundreds; and the number of distinct genotypes, from several hundred to 1,200 [13]. The overall number of phages and the ratio between the different species can change considerably during the course of time. Issues of whether certain types of phages are associated with certain animal species, and the biological geography of phages associated with animals and humans still remain obscure.

## Culturable bacteriophages in the normal microflora of animals and humans

Bacteriophages usually have a very narrow range of host specificity; the infectivity of each phage is usually limited by a specific range of bacterial strains, which belong to a single or several closely related species of bacteria. However, in certain cases phages can infect bacteria from different species and even different genera. F. D’Errelle (1921) first suggested that the isolation of *Yersinia pestis* phages from rat fecal matter 3 months after the end of a plague epidemic can be attributed to the growth of these viruses in bacteria of different species (in our view, it can be also explained by the persistence of *Yersinia * in rats). A recent study reported the persistence of vibriophages in oysters, in absence of the host during the winter period [30]; the authors suggested that vibriophages can use alternative host(s). Thus, the multi–species or multi–genus specificity of certain phages can be of ecological significance in the symbiotic microflora of animals. Nevertheless, even closely related strains of the same species can differ in their sensitivity to phages. This means that the effect of a phage infection on different populations of various species or strains can vary significantly. Cultural methods are currently the only approach for studying the ecological interactions between phages and their hosts at the strain level.

There is a large number of studies which report the isolation and characterization of certain bacteriophages, which are obtained from humans or animals. Bacteriophages of *Streptococcus bovis, S. durans, Prevotella bryantii, Bifidobacterium ruminale * were extracted from the rumen of various species of ruminant animals [14, 31]. Fecal matter from humans and various animals was used to isolate phages of *E. coli* , * Salmonella* , * Bacteroides * , * Klebsiella * and other bacteria [32–43]. In most cases, the phages were isolated using laboratory strains of the appropriate species, or using wild isolates obtained and characterized in advance during the course of a large body of studies which used phages as indicators of the fecal pollution in water.

The presence and titers of DNA coliphages vary considerably between different animal species and even between individual animals, which is consistent with the fact that a noncultivatable viral community is highly variable [44, 45]. However, there are no reports on the specific association of any types or groups of DNA coliphages with a particular species of animal. In contrast to this, the presence of F–specific RNA (F–RNA) coliphages ( * Leviviridae* family) in animal fecal matter exhibits a certain species–specificity. These bacteriophages can be divided into 4 genotypic groups, which can also be distinguished serologically. The occurrence of specific serotypes can vary considerably between different species of animals: horse fecal matter, for instance, rarely has any F–RNA phages, while more than 70 % of chicken droppings samples show high titers of these viruses (10^5^–10^7^ PFU × g^–1^). Only 10–20 % of human fecal matter samples contain F–RNA phages, but groups II and III are most often present in these samples (> 80 % from the overall number of isolates), as opposed to other animals, in which groups I and IV are found with about the same frequency [33, 39, 42, 46]. Currently, there are no satisfactory explanations of this type of association.

The detection of a possible association between several genetic subgroups in the known genera of tailed bacteriophages and their macro–host species may be complicated by the fact that simple and productive methods for the selective extraction of genetically related phages from wild samples have not yet been developed. Nearly selective isolation of phages related to T–even phages from the fecal matter of a patient suffering from pediatric diarrhea in Bangladesh was observed when using *E. coli* K803 strain as a test culture in [47]. Inoculation of a lawn of the enteropathogenic *E. coli* O127:K63 strain led to the isolation of a completely different set of phages, all of which were members of the *Siphoviridae* family.

## Study of enterobacterial indigenous phages

Furuse *et al*. [36] discovered that the amount of titers of coliphages in the fecal matter of healthy people is usually low and that the pool of free viral particles is usually formed by temperate phages. These data indicate that phage particles in the intestines of healthy people are produced mainly via spontaneous induction of lysogenic bacteria. Thus, the reproduction of phages in lytic cycle is of limited importance to the ecology of coliform bacteria in the human intestine. As opposed to healthy people, some patients suffering from internal diseases or leukemia exhibited an elevated number of titers of coliphages, and a major portion of isolated bacteriophages are virulent phages [46]. In several patients, the growth of coliphage titers correlated with the severity of the patient’s condition.

The data presented in [36] are in agreement with the fact that attempts to isolate coliphages from dog fecal matter by using indigenous strains of *E. coli* had no success [48]. Using more than 500 indigenous strains of *E. coli* as hosts, 6 samples of fecal matter from domestic dogs were screened for coliphages, but none were found. Only one of the samples yielded phages, which were active on the laboratory *E. coli* strain C. On the other hand, 16 dogs kept in an animal kennel were found to have various titers of coliphages from 0 to 10^7^ PFU × g^–1^. The authors suggest that the absence of phages in domestic dogs was due to their isolation from their kin and the “too clean” living conditions. Recontamination by fecal microorganisms is understandably limited for humans as well, which can partly account for the above–cited results [36].

The results of our recent study, which used horses as a model [15, 49], are in contradiction with the data in [46]. The celluloseolytic microbial community in the horse colon is highly complex and can include more than 500 species of bacteria, archaea, and also fungi and protozoa. In contrast to the rumen community, the microbial biomass of the colon is not subjected to further enzymatic hydrolysis and is excreted with the fecal matter in practically unchanged conditions. The physico–chemical conditions in the equine colon seem to be more stable than that of most other animals, since the average time of cellulose–rich food (grass) digestion is 72 hours, which is considerably more than the time windows between eating or defecation events [50].

In order to study the ecological interactions between coliphages and their hosts under these conditions, we monitored the dynamics of coliphage and coliform bacteria titers in 4 animals taking samples every 48 hours. Phage titer was measured using the laboratory * E. coli* test–culture C600. Study [36] analyzed 9 series of samples obtained from 19 healthy people at two week intervals and did not detect any significant temporal changes of coliphage titers or variety. In horses we observed significant changes in the phage titer (2–4 logarithmic units for different animals during the course of the 16–day observation period). However the titers of coliform bacteria were much more stable and fluctuated only slightly around 5 × 10^5^ CFU × g^–1^. The difference between coliphage dynamics in humans and in horses may be an indication of a more significant role played by phage infection in the ecology of the latter’s enterebacterial ecology.

We did not detect any correlation between the titers of coliform bacteria and coliphages in our animal group. This result may be due to the extremely high variety of * E. coli* strains and other bacteria which make up the coliform pool in horse intestines [51]. In order to differentiate between closely related isolates, which can however differ in their phage sensitivities, we employed a simple scheme of PCR–fingerprinting [49]. Using this approach, we demonstrated that the pool of coliform bacteria present in the samples of horse fecal matter includes up to 1,500 individual strains (estimated using the nonparametric Chao1 criterion). We also estimated the share of coliform strains which were sensitive to a certain phage isolate from the same sample. On average, about 1–8% of the strains turned out to be sensitive. The variety of bacteriophages which display activity towards a specific indigenous strain of *E. coli* obtained from the same sample is usually very low and is limited to 1–2 genotypes distinguishable by restriction analysis. The variety of phages which could be detected by plating on the laboratory *E. coli* strain C600 was also limited; only 1–3 types in each of the samples studied. It seems that this results reflect the severe competition of the viruses for the available host cells.

## Effects of viral infection on the bacterial populations of the GIT

Microbial biomass can account for up to 54% of the hydrated mass of human feces. [52]. Coliform bacteria (> 80 % of which are usually represented by *E. coli* strains) are usually present in human and other animal feces in titers of about 10^5^–10^ 8 ^;CFU × g^–1^ [39]. * Streptococcus bovis* is present in the ruminal fluid of sheep in concentrations of 10^6^–10^7^ CFU × ml^–1^ [14, 53]. These population densities are higher than needed for the exponential reproduction of phages (10^4^ cells × ml^–1^ for most phage–host systems) [54]. Thus, some mechanisms that stabilize the co–existence of phages and bacteria must exist.

We assume that coliphage infection is a selective factor which retains a high level of intraspecies variety of coliform bacteria in horse intestine limiting the chance for the few best competitors strains to outgrow the others. On the other hand, the high variety severely limits the availability of host cells for each specific type of coliphage, thus stabilizing the whole system. The high degree of variety and the reticular organization of the system make direct differential monitoring of individual strains and their associated phage populations very difficult, thus preventing direct validation of this hypothesis. However, we discovered recently that the intraspecies variety of coliform bacteria experienced a severe reduction after peroral application of enrofloxacin for a prolonged period of time, due to a severe leg wound to a horse. The overall titer of coliforms remained at normal level (the dominant strains were resistant to the antibiotic). Currently, we are developing the idea of using such animals with an artificial decrease in the intraspecies variety of symbiotic coliforms as a model for studying bacteriophage ecology, since it will allow us to perform direct experimental tests to prove our hypothesis. Another approach to this problem is analyzing the formation of a normal microflora in newborn horse foal.

The observed structure of the intestinal coliform bacteria and coliphage community is in agreement with the results of mathematical and experimental modeling of communities consisting of bacteria and their phages living under conditions allowing the co–evolution of both components [55–58]. The population of hosts exhibited an observable split into a multitude of genetic lines with varying phage sensitivities. In turn, the phages were selected for widening of their host variety, albeit at the price of absorption efficiency. Thus the initial two–part system became much more complex [57]. Interaction with bacteriophages can also facilitate the selection of bacterial strains bearing mutations of their mismatched base reparation genes, or other mutations that destabilize the genome [59, 60]. Such hypermutable strains have an advantage only in rapidly changing conditions, where the possibility of obtaining new traits counterbalances the accumulation of deleterious mutations [61]. The presence of bacteriophages which are able to evolve * in situ * seems to be one of the types of such a variable selective pressure. The presence of a large number of hypervariable strains in natural enterobacterial populations has been demonstrated [62]. Thus, phage infection can facilitate the increase in phenotypic variety not only by direct selection of resistant strains, but also by supporting the hypermutable strains in the population.

Bacteriophage infection can play a certain role during the events that triggered in the horse GIT when it was overloaded with carbohydrates. A hypothesis has been advanced that certain toxins produced by the altered microflora found their way into the bloodstream and somehow, either directly or indirectly, caused lowered adhesion of the hoof epithelium to the basal membrane. It was recently shown [63] that a rapid increase in the number of streptococcus of the * S. bovis / equinus * group in the colon takes place 8–16 hours after the administering of oligofructane. However, the number of these bacteria dropped very rapidly afterwards. One of the possible explanations is a massive infection of the streptococci by the appropriate phages, similarly to the phage control mechanism during cyanobacterial water blooming [10].

The significant selective influence of natural phages was demonstrated on a population of *Campylobacter jejuni* in chickens. The amount of * С*. *jejuni* phages in the chicken’s appendix was negatively correlated to the degree of colonization by bacteria (an average of 10^5^ CFU × g^–1^ in phage–containing samples, as opposed to 10^7^ CFU × g^–1^ in samples where no phages were found) [64]. It later turned out that the presence of phages selects variants of * C. jejuni * bearing extensive rearrangements of the genome [65], which occur because of horizontal transfer or as a result of intragenomic inversions with breakpoints in two Mu–like prophages integrated into bacterial DNA [66]. In both cases, the variants which were sensitive to the phages had a considerable advantage in the colonization of broiler chickens. In the absence of phages, the population rapidly reverted to the sensitive phenotype. The authors suggested that the genomic rearrangements in * C. jejuni* occurring in the GIT of the birds played the role of an adaptive mechanism that allowed the bacteria to survive the periods of phage activity and the consequent competition for resources.

## The effect of artificially introduced phages on GIT bacteria

Effective elimination of pathogenic bacteria from the GIT using phage preparations has been demonstrated in multiple experiments on the therapeutic use of phages [2, 7]. The therapeutic effect of the phages can be limited to a decrease in the pathogen’s population down to a point when the immune system can effectively control its reproduction. In some cases, even such low doses of phage as 10^2^ PFU could prevent the development of an infection by artificial introduction of pathogenic * E. coli* [67]. It is noteworthy that these low dosages were much less effective if they were administered after inoculation with the pathogen. This is an indication that a population of pathogenic bacteria is much more susceptible to phage attack before it has colonized certain protected regions of the intestine.

The impact of an artificially introduced phage cocktail was studied on resident and introduced populations of *E. coli* in mouse intestines [68]. The mice used in these experiments stably excreted * E. coli* at titers of about 10^6^ CFU × g^–1^ of feces. This value varied slightly during the course of time, but the presence of natural coliphages was undetectable on the indicator *E. coli* K803 strain, which was sensitive to all the phages used in later experiments. The mice were treated with a phage cocktail (titer 10^7^ PFU × ml^–1^) through their drinking water. These phages were summarily active against practically 100% of the resident strains, which were isolated from the animals before the experiment. Nevertheless, the impact of the externally introduced phages on the titers of coliform bacteria was insignificant, and the phages were incapable of sustained propagation by infection of the resident bacteria. The *E. coli* isolates obtained during the experiment exhibited the same sensitivity pattern to the components of the cocktail, which ruled out a shift of the resident population towards the prevalence of resistant variants. However, the phages did effectively lower the amount of a sensitive *E. coli* strain that was introduced into gnotobiotic mice a week prior to the administering of the phage cocktail. This effect was accompanied by active replication of the phages in the intestine. However, the surviving portion of the population remained sensitive to the phages, which indicates the existence of certain niches in the mouse intestine where the bacteria are either physically or physiologically shielded from the phage infection. Since practically all of the resident population of normal mice is protected, these “shelters” can accommodate almost all the *E. coli* population in the mouse intestine by *E. coli* . Interestingly, mice obtained from the same animal care facility a year after the experiments had practically no *E. coli* in their intestines and were colonized by other species of enterobacteria [Br ü ssow, personal communication]. This observation is in agreement with [69], which found that less than 20% of the 48 mice studied in the project bore resident *E. coli* in their GIT, while coliphages were present in minimal titers in those cases. The author suggested that one of the main reasons blocking the reproduction of coliphages in the GIT of mice is the absence of a sufficient population of hosts.

The production of phages in the intestine is also affected by the physico–chemical factors in the environment. Bile salts and carbohydrates, for instance, can have an inhibitory effect on the absorption of many coliphages [70]. This effect is negated by the expression of the Ag43 protein, which is a bacterial surface adhesin that promotes cell aggregation, for instance during the formation of biofilms. Ag43 synthesis is controlled via the phase–variation mechanism; thus, a phage infection can sometimes screen for cells that have a reduced ability to form biofilms. Notably, some phages of *Bacteroides * exhibited an opposite reaction to the addition of bile salts to the culture media, growing more effectively [71].

Growth in a biofilm on the surface of a mucous membrane or on food particles can play a major role in containing viral infection of bacteria in the GIT. Some data [72] seem to indicate that *E. coli* populations in the mouse’s intestine are in great part made up of cells in a state of starvation, which are physiologically ill–suited for phage reproduction. The actively reproducing share of the population may be located only in microcolonies on the mucous membrane of the intestine [73]. However, both of these studies used mice treated with streptomycin and artificially inoculated by a single * E. coli* BJ4 strain with a known phenotype. Moreover, the fact that the digestion processes of mice can vary significantly from those of larger animals is material. The metabolic rate of mice is much higher, and the food travels through the intestine much more quickly than in horses or humans. Furthermore, the ratio between the mucous membrane’s surface and the volume of the intestine is considerably higher, which facilitates a much more rapid uptake of nutrients. Thus, the issue of to what degree can mouse–obtained data be extrapolated onto larger animals remains to be settled.

## Regions of the body exhibiting lowered phage influence on the microflora

Current data suggest that phages can, at least in some animals, successfully overcome the physico–chemical barriers that prevent infection and effectively reproduce using resident hosts, thus constraining their populations. Taking into account the openness of the GIT ecosystem in terms of matter exchange with the outside environment, the significant role of bacteriophages comes as no surprise. However, the situation can be significantly different in other ecosystems of the organism, which are also densely populated by microbiota. Hitch *et al*. [29], for instance, could not isolate from the human oral cavity any phages that proved active towards indigenous bacteria, which made them conclude that the bacteria in that system are not significantly affected by phage infection. A satisfactory explanation has yet to be given to account for this fact.

Some species of bacteria are abundant in the microbial system of ruminant animals. *S. bovis* can be present in concentrations of 10^6^–10^7 ^;CFU × ml^–1^ [14, 53]; moreover, the whole *S. bovis* population of a sheep’s rumen can belong to the same phagotype [53]. The authors observed a rapid shift from the dominant strain to a different one, with a different phagotype, but these processes were not coupled with the appearance of any bacteriophage able to lyse any of the above–mentioned strains in the rumen fluid [53]. Nevertheless, the presence of *S. bovis * phages in the rumen in titers of 10^1^–5 × 10^4^ PFU × ml^–1^ variable in different individual animals was later demonstrated [74].

The ecology of ruminal streptophages was thoroughly studied by Tarakanov and coauthors in the 1970s–1980s. Sadly, most of the results published by these authors are contained in hard–to–access journals and government reports, which is why we reference a book [14] that has a detailed review of these studies. The authors detected *S. bovis* phages in the rumens of cows and sheep in titers of 10^1^–10^4^ PFU × ml^–1^. The results of carefully controlled field experiments showed that the peroral introduction of an *S. bovis* phage preparation (streptophagin) into cows led to an increase in the phage’s concentration by a factor of 10^4^–10^5^ , accompanied by a decrease in the amount of amilolytic bacteria, a decrease in the amilolytic and increase in the cellulolytic activity of the ruminal contents. These changes led to improvement of yield and quality of meat and milk produced by the treated animals. This was confirmed by detailed and well–documented research [14]. After the discontinuation of streptophagin, the phage titers returned to their initial values fairly quickly and the amount of amylolitic bacteria increased. These results were an indication that a preparation of a single type of phage could have a considerable impact on a large portion of the *S. bovis* population in rumens. This is in agreement with research [53] which demonstrates the low intraspecies variety of *S. bovis* in sheep rumens. On the other hand, it is obvious that an unknown factor prevents the effective reproduction of streptophages in the resident bacterial population in the system.

It was shown that tannic acid can inhibit phage reproduction in physiological concentrations by causing the precipitation of phage particles [75]. Tarakanov [14] has demonstrated that the ruminal fluid of cows and acetic acid at physiological concentrations can both inactivate * S. bovis* bacteriophages. Notably, the sensitivity of different phages varied considerably and was dependent on the concentration of the inactivating agent used. Inhibition of phage activity in the rumen by various chemicals can play a major role in the control of the phage lysis of resident cells. This model is in agreement with the conclusion in study [76], which declares that the sometimes–observed mass lysis of bacterial biomass in the rumen is most probably a result of autolytic processes as opposed to phage infection.

The female and animal vaginas are also an ecological niche extensively colonized by bacteria. The bulk of the human vaginal microflora is made up of lactobacilli. Species and strains of these bacteria vary among individuals [77], but normally each individual woman has 1–2 dominant strains [78]. The colonization density is relatively high and is about 10^6^–10^7^ CFU per vaginal swab. Thus, it can be assumed that this population may be susceptible to mass lysis by bacteriophages. Yet all attempts to detect free phages in vaginal excretions have been unsuccessful [79], even though lysogenic lactobacilli strains are often found in the vaginal microflora [79, 80].

Even though the vagina is less susceptible to bacterial and viral exchange with the environment, excluding the exchange associated with sexual intercourse, studies of vaginal swabs of clinically healthy women often show an unexpected drop in the population of lactobacilli. In most cases, the normal flora regenerates on its own, but some individuals develop the so–called anaerobic bacterial vaginosis, a disease during which the lactobacilli are replaced by anaerobic bacteria, such as * Gardnerella vaginalis* , * Prevotella, Porphyromonas, Atapobium * and * Mobiluncus* *species * . Epidemiologically, the disease seems to be a sexually transmitted disease [81, 82], even though the etiological agent has yet to be identified. In [81], A. Blackwell suggested that the reason for this disruption in lactobacilli flora, which in some cases leads to vaginosis, is a phage infection. We hypothesize that this can be the case when the vagina is infected by lactobacilli lysogenic with respect to a phage active in the resident strain.

Lysis of host cells by moderate phages is usually pretty infrequent: 10^–1^–10^–2^ [83–85]. Thus, the reproduction of these viruses in an ecological niche densely populated by the sensitive host strain usually happens via the lytic scenario, causing the death of the majority of resident cells. The latter may then be replaced by the descendants of the lysogenic strain which initially released the phage, and by newly lysogenized bacteria or by other bacterial species. Such a scenario was modeled mathematically and experimentally [83] on a model population of *E. coli* . Bearing in mind that the frequency of a spontaneous induction of most lysogenic strains of vaginal lactobacilli is less than 10^–8^ PFU × cell^–1^ [79] in lab cultures, any factor that increases the induction frequency can increase the chance of an “ecological catastrophe” in the vaginal microflora. This can explain the strong epidemiological correlation between smoking and the risk of bacterial vaginosis [81], since some substances of tobacco smoke can cause induction of prophages in lactobacilli [86]. However, the results in study [80] contradict Blackwell’s hypothesis [81]. The authors showed that lactobacilli prophages often lose their ability to induce and to reproduce in cells, seemingly because they are selected in the presence of hydrogen peroxide, which is produced by many bacterial strains and is an activating agent for SOS–inducible prophages. A detailed study of the temporal dynamics of lactobacilli strains in the vagina and of the lysogeny of these populations may provide the key to understanding the phage nature of vaginosis cases.

## Phage–cell interactions, ecologically significant effects

In many cases, isolates of enterobacteria (and probably other groups of microorganisms) obtained from the wild are partially resistant to bacteriophages, which coexist with them. This resistance can manifest through a lowered inoculation efficiency of a specific phage, slow absorption, or a decreased yield of the phage, which may be due to the effects of the restriction–modification system [59, 87], and also of a whole range of specific resistance systems developed by bacteria to counter some groups of phages [59, 88, 89]. Moreover, prokaryotes often have CRISPR–loci (clusters of regularly interspersed palindromic repeats) [90], which contain short sequences extracted from the bacteriophage genome, plasmids, or other alien DNA by an as–of–now–unknown mechanism. The genes associated with CRISPR encode for proteins that form enzymatic complexes that can attack RNA or DNA molecules that include sequences identical to the so–called CRISPR spacers [91, 92]. Such systems allow bacteria of numerous species to rapidly develop resistance to a specific phage strain, due to a decreased efficiency of infection. This resistance can however be circumvented by point mutations of the attacked sequences in question [28]. Quantative data that characterize the frequency and ecological significance of the systems that provide resistance to phages in microbial communities by affecting the intracellular development of the virus is still missing. This issue deserves special attention and study, since the mechanisms that cause abortive infection turn a portion of the cells that can absorb the phage into ecological traps, which accelerate the rate of decay of free phage particles considerably [94].

In a recent study by our group, only 1–4% of the coliform bacteria strains isolated from horse feces samples exhibited sensitivity after cultivation on phage agar prepared with phages extracted from the same series of samples from the same animal [49, Tarasyan, unpublished data]. However, these phages showed a considerably decreased inoculation efficiency (10^–2^–10^–4^) on lawns of some isolates [Tarasyan *et al* , manuscript in preparation]. In some cases, this effect is due to the ineffective absorption of the phage and can be circumvented by mutational or recombinational rearrangements of the phage’s adsorption apparatus. Interestingly, even closely related bacteriophages extracted from the same animal can have slightly different host ranges [49]. This seems to indicate the high * in situ* adaptation rate of the absorption apparatus. Thus, the level of isolation existing in the phage–host system can be severely underestimated. As a result, the populations of each distinct genetic line of phage exist in conditions of strictly limited numbers of available host cells. In some cases, the effective concentration of phages and their respective sensitive cells drop to such a low level that one can expect the extinction of the phage population. For instance, the concentration of bacteria in a horse feces sample in our example mentioned above was 10^3^–10^4^ CFU **×** ml^–1 ^[49, Tarasyan *et al* ., unpublished data], which is below the minimal value needed for effective phage reproduction [54]. The concentration of the corresponding virulent phage was approximately 1 0^2^–10^5^ PF U × ml^–1^, and this strain of phage, which can be identified by its restriction profile, persisted in the intestine of this animal for more than 2 years [our unpublished data]. The mechanisms that stabilize the long–term coexistence of this virus and its hosts in the intestinal ecosystem remain unclear. Obviously, any adaptation of the bacteriophage that leads to an increase in the range of potential hosts will provide an immediate selective advantage.

It is thought that some phages can carry “secondary” adhesion proteins which are not directly connected with the apparatus triggering the DNA ejection . Many of these putative secondary adhesins contain immunoglobulin–like domains which are exposed on the surface of the heads, contractible covers of the tail, or on the ends of the collar fibers of the phages [95]. Some phages can carry domains on their collar filaments, which are similar to adhesins not related to immunoglobulins [96]. Data on whether such structures can affect the growth of phages in laboratory conditions is still missing, but in an ecological situation such as the one prevailing in an animal intestine, their role can be considerable. First of all, it can be achieved by increasing the probability of reversible absorption after a phage particle has met with a sensitive host–cell. The increased absorption rate is not beneficial in some ecological situations. When phages grow on a dense population of hosts immobilized on a viscous agar medium (for instance when cultured using the double–layer method), mutants that have a lower adsorption constant have a considerable advantage and form plaques of larger size and with a higher number of virus particles [97]. This is due to the increased rate of the viral diffusion into the viscous media filled with host cells, and into the surrounding liquid. The authors suggest that similar advantages for slowly absorbed phages can be important during development in biofilms and during the spread to new regions. The mechanisms that lead to the reversible loss of secondary adhesins (for instance some of the tail fibrils of many phages) can thus have a significant effect on the adaptation of phages to growth on bacterial biofilms or on plankton cells [97]. Notably, secondary fibrillar adhesins can limit the diffusion of viscous media in several ways, including through an increase of the effective radius of the virus particles [97]. From this, it follows that preparations of phages obtained by cultivation in liquid or viscous media can be enriched with genetic variants optimal for the given mode of growth of the host population. The phages that form larger plaques may be less effective during growth in liquid media, since they exhibit a decreased level of absorption. These ideas should be taken into account when developing therapeutic phage preparations.

The importance of lysogeny in the ecological interactions between phages and bacteria in symbiotic microbial communities of animals requires further study. Some data [36] suggest that the induction of lysogenic bacteria is the main source of free coliphage particles in the intestine of healthy humans. However, most moderate coliphages isolated from the free phage particles pool of a sample of human or animal feces were members of the lambdoid phage group, while phages obtained by induction of lysogenic strains were mostly of the P2–like group [98]. In our recent study, all the phages extracted from horse feces and placed into one of the two groups by analyzing particle morphology and sequencing random portions of the genome turned out to be professional–virulent phages [49, our unpublished data]. It is impossible to tell at this point whether this discrepancy in the results is indicative of a difference in the microbial systems of animals or is an artifact of the research method.

Coliphages extracted from feces can often form metastable associations, which can be cultivated in laboratory conditions for several passages. We have termed these pseudolysogenic associations [A. Zimin, personal communication; our unpublished data]. The mechanisms which stabilize such associations are not always understood [99]. The most probable explanation is that some portion of the cells in a colony is partially shielded from phages by polysaccharide material or due to the physiological condition of the cell. In some cases, phages and bacteria can diverge during propagation on a agar medium, turning the pseudolysogenic association into a complex multicomponent community [our unpublished data]. Additional research is needed to understand whether pseudolysogenic associations of virulent phages and their hosts play a significant role in the * in vivo* ecology of coliphages or whether this is only a laboratory phenomenon.

## The pharmacokinetics of phage preparations

**Penetration of bacteriophagesinto the internal environment of the organism**. It is known that bacteriophages can penetrate into the bloodstream from the intestine and other regions of the body, which are inhabited by microorganisms, and then be transported by the blood flow [100, 101]. It seems that the presence of a certain number of natural bacteriophages in the blood is normal (the term “physiological viremia” was suggested for this phenomeneon) [101]. A recent study of the pharmacokinetics of three different phages on chickens [102] showed that peroral introduction of phage preparations led to the appearance of only one of the three phages in the liver and spleen in small quantities. This phage was also present in the lungs; however, it could be accidently inhalted by the birds since the phage preparation was instilled by a syringe into the mouth. So, this begs the conclusion that this way of administering the suspension does not lead to effective translocation of phages, despite the fact that this study used relatively high doses of phages (10^6^, 10^7^ and 10^8^ PFU per individual). Phage administration was slightly more effective in aerosol form through the lungs. However, earlier studies [103–106] have demonstrated multiple cases of the effective introduction of therapeutic phages into the blood using rectal and peroral administration. Peroral use of the “Sextaphage” preparation (FSUE “Microgen”, Russia) yielded detectable bacteriophages in the blood an hour after administration [105]. Similar results were obtained when using this preparation as a rectal suppository [105, 106]. These studies do not report the phage dosages, listing only the volume of the preparation. They also do not report the phage titers in the blood, and there is no data on the differences between the penetrations of the various phages from the complex preparation. Notably, the rate of phage translocation from the GIT to the blood stream (similarly to bacteria) can vary significantly depending on the physiological conditions. An inflammation considerably increases the translocation of bacteria (and probably phages) from the intestine [12]. It is not yet known which specific phage traits affect their translocation through the mucosa. Probably, the transport is receptor–dependent and is executed as an active process by specialized cells of the immune system ( М –cells, goblet cells) and possibly by epithelial cells of the intestine and other components of the GIT. Several studies have attempted to identify the amino acid sequences that are involved in the binding with the appropriate receptors. These studies used the phage display approach [107–110]. Libraries of random peptides exposed on the surface of the filamentous М 13 phage (which normally exhibits ineffective translocation through the mucous membranes of the GIT) were introduced into the GIT of experimental animals, after which the phages were extracted from the blood and internal organs or from the appropriate tissues from the GIT itself, and the peptides that were exposed on their surface were sequenced. The results showed that peptides YPRLLTP [108] and CSKSSDYQC [110] facilitate phage transport, peptides lettcaslcyps and vpphpmtyscqy [109] enhance the binding to М –cells and to rat Payer patch tissue, and the lthpqdsppasa peptide stimulates the binding of the phage particles to the mucous membrane of the mouse intestine, which was damaged as a result of a strong systemic inflammatory reaction [107]. As opposed to the cited results, study [111] identified no significant effect of 7–amino acid peptides on the transport of the M13 phage from the mouse intestine into the bloodstream. It is quite possible that this result is due to the specifics of the experimental system used by the authors, or that 7 amino acid residues are not of sufficient length for the effective binding with appropriate cell receptors on the intestinal mucous membrane of mice. Currently, there is data in the literature on attempts to select therapeutically promising phages (mostly so–called tailed phages) for their ability to effectively travel into the bloodstream from the GIT. The success in selecting the phages that can circulate in the blood for prolonged periods of time (see further) gives hope that this procedure can be effective in some cases. Notably, numerous results indicate the high efficiency of phage translocation into the blood during rectal administration of the preparation [103, 105]. This phenomenon is being exploited in therapeutic practice now. There are phage preparations on the Russian market that are sold as rectal suppositories (see above); however, the mechanisms of phage translocation from the rectum and the factors that influence this process deserve a more thorough analysis.

Intramuscular or intraperitoneal administration of phages leads to much more effective translocation into the blood and organs than peroral methods. Thus, data from [102] shows that 3 hours after intramuscular injection of 10^8^ PFU of three types of phages into chickens, their concentrations in the spleen were 2 ** × ** 10^2^– 3 ** ×** 10^4^ PFU **× ** ml^–1^ and 6 ** ×** 10^2^ – 10^4^ PFU **×** ml^–1^ in the liver. During the following hours, the phage concentration dropped rapidly, and after 24 hours the phages were practically eliminated. Of note were the interesting differences in the effectiveness of infiltration of bird organs by different types of phages injected at the same doses. Intraperitoneal injection of 10^11^ PFU of phages (4 phages were tested) into mice resulted in a blood concentration of 5 ** × ** 10^8^–2 ** × ** 10^10^ PFU **× ** ml^–1^ two hours after injection, which indicates the effective infiltration of the inner environment of the organism [112]. One of the earlier (but also one of the most rigorously performed) studies of the phage pharmacokinetics [113] showed the effective infiltration of * Shigella dysenteriae * bacteriophages through the mouse’s hematoencephalic barrier. Intraperitoneal injection of ultrafiltration–purified lysates with phages led to an almost immediate appearance of phages in the blood. Infiltration of healthy animal’s brain tissues was recorded only in a few individuals on a small scale. On the other hand, mice infected with * S. dysenteriae * (injected intracerebrally with a dose exceeding DL95 %) showed a rapid increase of the phage titer of up to 10^7^–10^9^ PFU **× ** g^–1^ in the brain tissue. During the following monitoring period, the phage titer in the brain decreased slower than that in the blood. The survival rate of mice that received 10^7^–10^9^ PFU of the phage was 72 % as opposed to 4 % in the control group, which received a heat inactivated phage preparation.

## The distribution of phage particles in the organism and their elimination

Early research in the 1920s–1930s [2] demonstrated that bacteriophages are eliminated from the bloodstream very rapidly. Mice that received intraperitoneal doses of phage [112] had very quick increases in blood phage titer (up to 10^10^ PFU × ml^–1^), after which the titer decreased approximately by a factor of 10^3^ after 12 hours and continued to decrease in a soft curve. The authors approximated this two–phase curve using the following equation: С = 3.525 ** ×** 10^9^ **× ** e^–0,753t ^+ 2.35 ** ×** 10^7^ **× ** e^–0,0997t^, where С is the concentration expressed in PFU **× ** ml^–1^; t is the time, in hours; while the numeric parameters varied for the different types of phages. Some bacteriophages can be absorbed onto erythrocytes and leucocytes in the bloodstream [93, 114]. Thus, it is unclear if such sequestered phages can infect the targeted bacteria during phagotherapy. Phage particles are most actively absorbed by the liver, where they quickly decay [102, 115]. Phages survive slightly longer in the spleen [2, 102, 116]. It seems that the reticuloendothelial system plays a major role in this process as opposed to the circulating phagocytes, since mice with cyclophosphamide–induced neutropenia exhibit only limited inhibition of phage elimination [112].

Phage particles are subjected to effective elimination by the reticulo–endothelial system. However, it is possible to select phage strains with altered surface proteins, which exhibit a significantly increased half–life in the bloodstream (one amino acid substitution may suffice [117]), and such long–circulating mutants can remain in the bloodstream for up to more than a 1,000 times longer than the initial strain [117–119].

Excretion of phages with urine has been shown in many cases [104–106, Darbeeva, personal communication]. However, this mechanism does not seem to play a major role in the phage elimination process, since the concentration of viruses in urine is approximately 10^1^–10^2^ [106], and such excretion is insignificant. On the other hand, even this insignificant number of bacteriophages, which make their way into the urinoexcretory system, is sufficient to have a therapeutic effect in the treatment of urological infections by peroral administration of phage preparations [104]. It is safe to assume that inflammation–mediated damage to the basal membrane of the Malpighian tufts can lead to increased phage excretion. It is also possible that the phages find their way into urine not via filtration through the tufts, but also through transfer from the blood into the canal epithelium. The issue of phage excretion with urine and the factors that regulate this process require further investigation.

The production of antibodies specific to bacteriophages in patients can block the positive effect of phage therapy. Moreover, healthy people and patients who have never undergone phage therapy also have a certain level of antiphage antibodes [120]. However, the available data shows that the presence of such antibodies has a weak correlation with the results of therapy [A. Gorski, personal communication, 2]. It is worth noting that not all of the antibodies which interact with the phage particle have a direct neutralizing effect. In order to have this effect, antibodies would have to interact with adhesins and, in the case of some phages, with other elements of the tail. The production of specific high–affinity immunoglobulins of the IgG class takes several weeks; that is why this problem is of little significance for short courses of phage therapy. Moreover, the immunogenic potential of various phages is very different, and some bacteriophages require the use of adjuvants and repeated immunizations in order to achieve a significant immune response. It is also worth noting that the amount of antigens in the therapeutic doses of purified bacteriophages is relatively low; for instance, 10^10 ^particles of Т 4 phage contain only about 1 microgramm of protein (this calculation is based on the assumption that the mass of DNA and protein is approximately the same in tailed phages [121]).

## Immunobiological activity of phage particles

The direct effect of certain phage particles on the immune systems of humans and animals has been demonstrated recently by a research group led by prof. Gorski * . * It was shown that the viral particles of T–even bacteriophages lower the amount of the reactive oxygen species produced by neutrophils, which were induced by bacteria or endotoxin, and suppress the activation of T–cells, thus facilitating transplant tolerance. These phages also had a degree of antitumor effect [122–124]. The use of bacteriophage preparations in the treatment of various infections associated with * Staphylococci * and * E. coli* was often accompanied with a significant drop in the amount of serum C–reactive protein, which is an important marker of inflammation. It seems that such a decrease cannot be explained only by the antibacterial effect of the phages and must be connected to the immunomodulating activity of the phage particles [5].

It was suggested that the binding of Т 4 particles to eukaryotic cells is mediated by the capsid apex protein gp24 (gene product), which contains the KGD amino acid motif related to the RGD motif recognized by β 3 integrins [101]. This binding can partially account for the immunosupressing and antitumor effect of the Т 4 phage * in vivo * and * in vitro * [125]. A mutant of the Т 4 phage, which has no gp hoc capsid decorating protein, has an increased immunobiological activity [125], which is possibly due to the partial masking of the β 3 integrin–binding motif by these proteins. Some phages have surface proteins that are structurally similar to the molecules involved in the signaling cascades of immunocompetent cells. A whole range of structural proteins of various phages have immunoglobin–like [95] or collagen–like domains [126]. Study [24] noted the considerable increase in the concentration of phage particles on an ulcerated intestinal mucous membrane and hypothesized that the immunologic activity of phage particles can play some role in the pathological process in the case of Crohn’s disease. On the other hand, it is thought that natural phage populations can take part in the interaction between the lymphoid tissues of the intestine and the microflora; for instance in the suppression of the inflammatory effect caused by massive contact with an antigen [101]. However, as of today, there is no experimental data that characterize the interaction of the natural viral community with the immune system.

## Dynamics of phage–bacteria interaction during phage therapy

Physicians and microbiologists, who are not well informed on the theoretical basics of bacteriophage ecology and phage therapy, often hold the misguided opinion that the appearance of even a limited number of phage particles specific towards the target strain of pathogen in the area of the body colonized by these bacteria will trigger rapid phage reproduction, which will continue until the whole bacterial population is destroyed. These views are even expressed in some methodological literature (which we do not reference for ethical reasons). However, the coexistence of phages and bacteria in the same ecotope is an often–seen phenomenon in nature [10], and it has been around since before the separation of eukaryota, bacteria, and archaea [11]. Moreover, the interaction between bacteria and their viruses is not always antagonistic on the population level. Furthermore, the activity of bacteriophages can lead, under certain circumstances, to increased variety and overall metabolic activity of natural bacterial communities [10].

Currently, the standard procedure in phage therapy is to use only virulent phages; meaning those that can exclusievly reproduce in the course of a lytic cycle. During such a course of events, each infected cell is lysed after a certain period of time, called the latency period, releasing 50–200 and sometimes more phage particles. This results in an increase in phage particle concentration. In natural systems, however, the viral reproduction process is constantly countered by viral particle destruction and their elimination from the studied system. In the case of phage therapy, the major factors are phage consumption by the reticulo–endothelial system, their sequestration through association with erythrocytes, tissue cells or the extrarcellular matrix, their destruction as a result of adsorption on dead or specifically resistant cells (see above), as well as excretion from the organism with urine and feces. It is also worth noting that the reproduction of phages is usually localized in the infection center, while the spread of the phage’s descendants occurs throughout the whole organism. If the bacteriophage’s * in situ * reproduction rate is higher than the rate of the spread and (or) destruction, then the phage concentration will increase up to the depletion of the available host cells. This is called active phage therapy [127], and it is probably the working scenario during phage therapy of certain intestinal infections, including experimental ones. In these cases, one or several doses of phages are enough to cure the infection [2, 6, 7]. Cases where the concentration of phages must be maintained in order to suppress the growth of bacteria (see further) and large quantities of phages must be introduced artificially, such as during the treatment of chronic diseases, are termed passive phage therapy [127]. Many real–world cases are combinations of these two scenarios.

Let us discuss the factors affecting the kinetics of the interaction of the bacterial population with the phages during therapy. The limiting step in the lytic cycle of the bacteriophage is the adsorption of the virus’ particle on the surface of a bacterium, which usually results in the injection into the cell of the viral nucleic acid (for therapeutic phages, this is genomic DNA). The absorption process is mediated by highly specific recognition of the appropriate receptors on the cell’s surface by phage adhesins [128]. These receptors are usually liposaccharides, surface proteins, teichoic acids, the components of the bacterial capsule, and other surface structural elements of bacteria. This recognition is a passive physical–chemical process, which is dependent on the collision frequency of phage particles and cells during Brownian motion [129]. This process is described by a kinetic equation of the second order, which means that its rate depends on the concentrations of the bacteria and the phage. Specifically, ln(P/P_0_) = e^–kCt^, where Р is the amount of phage particles adsorbed up to the time point “t”; С is the concentration of bacterial cells, which does not change with the passage of time t; and k is an absorption constant (ml × min^–1^). All the differences in the phage diffusion, number of receptors on the cell’s surface, inhibition of absorption by the physico–chemical factors of the medium, etc. are all factored into the value of “k,” which is measured empirically for each case [129]. Under laboratory conditions, most tailed phages in liquid media have a k value of about (1.5–2.5) ** × ** 10^–9^ ml × min^–1^.

It was discovered empirically that different phage–host systems have threshold values of host cell concentration, which are needed for “triggering reproduction” of the virus [54] (usually about 10^4^ CFU × ml^–1^). In real natural ecosystems (including animal organisms), the host concentration threshold needed to trigger phage reproduction ( МТ ) can be calculated as the concentration at which the number of phages absorbed during the time of the elimination of half the phages (t_1/2_) minus the length of the latency period (t_lat_) is enough to provide offspring numbering half of the initial population. Therefore, it is a cell concentration (C), for which P _( t 1/2– tlat )_ = 0.5 P_0_/Y , where Y is the yield of the phage per infected cells in these conditions (the death of some infected cells before the release of phage progeny must be factored into this value). When C exceeds the threshold MT value, phage production will exceed its elimination and, thus, the phage concentration in the system will increase.

There is also a threshold value of phage concentration С _ phage _ , which inhibits the growth of the bacterial population if exceeded [127]. For this value of C_phage _(IT), half of the bacteria will become infected during the time of an average generation (under the condition that the phage concentration during this time remains constant). The growth of rapidly reproducing bacterial cultures is inhibited under laboratory conditions at phage concentrations of about 10^7^ [127, 129];PFU × ml^–1^. This value has no practical importance for laboratory use, since * in vitro * С >> MT, and the rate of phage particle destruction can be ignored; thus viruses accumulate very rapidly, causing practically complete lysis of the bacterial culture. It is obvious that the *in vivo * use of phage preparations will create other ratios between these processes, which is why populations of pathogenic bacteria are rarely destroyed completely during phage therapy. Thus, it makes more sense to speak of reducing the numbers of the pathogen to levels at which the infection can be controlled by the immune system [127].

In real–world phage therapy, most bacterial populations colonize only specific niches in the organism, and a major part of the bacteria are in physiological states, which do not allow reproduction of phages, for instance in biofilm form [130]. Cells that inhabit a biofilm are in a condition different from that in a suspended culture [131], and the cell’s sensitivity to phages is lower [119, 132]. This is partially due to the limited diffusion in the matrix surrounding the bacteria in biofilms. Neverthelss, bacteriophages can infect cells that inhabit biofilms [132, 133]; some viruses encode enzymes which can hydrolyze the matrix polysaccharides. Moreover, the appropriate genes can be introduced into the phage genome by genetic engineering [134]. Notably, phage infection causes disruption in the structure of the biofilm, making the surviving cells more available to the immune system. Bacteriophages seem able to infect persister cells [135], which are one of the major causes of failure in antibacterial chemotherapy of such infections [136]. The spatial heterogeneity of a biofilm’s structure [131], and many other microenvironments in the human and animal organisms, can, as it seems, limit the spread of phage infection. Dead and stationary cells also restrict the phage expansion, since they can absorb phage particles. Thus, an active process at the micro–level is coupled with a passive process at the level of the whole organism. This effect can help explain the success in phage therapy of a range of infections; for instance pseudomonal osteomyelitis [ М . Kutetaladze, personal communication], which cannot be effectively treated with antibiotics not because the pathogen is resistant, but because of the specific localization of the infection. Even though the infiltration of phages into the center of infection is limited, the possibility of local infective chain reactions is probably the major cause of success in the therapy. Moreover, stationary cells with reduced physiological activity can be infected, even though they do not allow the phage to reproduce. The infection will then be delayed until these cells are activated [135, 136]. Thus, the combination of active and passive phage therapy is the most probable scenario during the treatment of stable chronic infections. Such treatment requires prolonged courses of therapy, which can lead to patients being cured [5, 6]. Such cases require maintaining the phage concentration in the infection center for a prolonged time by administering new doses. The treatment of wound infections can be performed by using drainages with a constant flow of the phage preparation into the wound, or by using preparations that release phage particles slowly, such as the drug “Phagebioderm”, designed in the G. Eliava Tbilisi Institute for Bacteriophages, Microbiology and Virology [137].

It is obvious that phage–resistant mutant bacteria can emerge during phage therapy. However, it is important to note that we could not find any documented case of unsuccessful phage therapy caused by the emergence of phage–resistant mutants. This can be explained by the fact that such mutants often have altered cell surface structures which act as receptors for the appropriate phages. The physiological price of such resistance is usually reduced growth [10, 138], colonization ability [7, 65, 66, 139, see also section 3.3], and virulence [139].

## CONCLUSIONS

The accumulating genomic and structural data [11] point to the fact that bacterial and viral coexistence has been continuing for more than 2 billion years. The “rules of the game” that have emerged during this time are such that the presence of phages in natural systems not only has no negative effect on the variety and overall activity of the bacterial community, but also increases them considerably [10]. As more and more becomes known about the biology and ecology of bacteriophages, it becomes more apparent that the seemingly simple idea of using the “natural enemies” of bacteria to fight infection, which was first tried in practice in 1919 by Felix D’Errelle and by prof. Victor–Anri Hutinel [2], is a clever trick that should allow the complete suppression of one player (the bacterial population) and thus result in the elimination of the other player (the bacteriophage population). In order to achieve this goal through a rational approach (as opposed to an empirical one), it is important to understand the mentioned “rules of the game” in the ecological systems that are part of the human and animal organisms.

The analysis of the specifics of phage ecology allows to understand the additional requirements for therapeutic phages (other than their host range and * in vitro* efficiency) that could help identify the directions phage therapy research ought to take in the 21^st^ century. Among these promising directions, we can mention 1) improving the technology of therapeutic phage selection, including developing and standardizing quick moderate–virulent tests, creating systems for typing potential therapeutic phages in order to allocate them to one of the known groups, as well as developing a methodology for analyzing phage genomes in order to asses their use in phage therapy; 2) creating databases and collections of characterized therapeutic phages, which can be used to quickly formulate individualized phage cocktails adapted to the needs of an individual patient; 3) developing methods to control the translocation and spread of phages in the organism, including methods for providing long–term circulation of phages in the blood, and their infiltration into various tissues and organs, etc.; 4) developing methods for testing the infective potential on bacteria which are in various physiological conditions or which colonize various “shielded” ecological niches in the organism, such as resident bacterial populations in the intestine, bacteria inhabiting biofilms, etc., developing methods for modifying these phage’s traits, including methods involving genetic engineering; 5) monitoring and controlling the immunobiological activity of phages and targeted use of the ability of phage particles to interact with immune system cells; 6) using certain bacteriophage proteins as therapeutic agents, such as phage lysines [140] and bacteriocins.

Moreover, further studies of the role of bacteriophages in the homeostasis of normal microflora and in the development of pathological phenomena may help identify new approaches to the therapeutic control of these processes.

## ABBREVIATIONS

GIT: gastro-intestinal tract
PFU: plaque-forming unit, which corresponds to a one viable bacteriphage particle, if the efficiency of infection in these conditions and in this strain is close to 1
CFU: colony-forming unit, which corresponds to a one viable bacterial cell
